# Human West Nile Virus Infection, Catalonia, Spain

**DOI:** 10.3201/eid1207.060164

**Published:** 2006-07

**Authors:** Domingo Bofill, Cristina Domingo, Neus Cardeñosa, Joan Zaragoza, Fernando de Ory, Sofia Minguell, María Paz Sánchez-Seco, Angela Domínguez, Antonio Tenorio

**Affiliations:** * Hospital de Tortosa Verge de la Cinta, Tarragona, Spain;; † Instituto de Salud Carlos III, Madrid, Spain;; ‡ Catalan Health Department, Barcelona, Spain

**Keywords:** West Nile virus, serosurvey, flavivirus

**To the Editor:** West Nile virus (WNV) is a mosquitoborne flavivirus that is widespread in Africa, the Middle East, Asia, and southern Europe, where it causes outbreaks and sporadic cases of the disease. It has become an emergent disease in North America, where it was detected for the first time in 1999 and became epidemic shortly thereafter (*1*). Although WNV was initially considered to have a minor health effect in the Mediterranean basin, human and equine outbreaks reported in the last decade in different countries (*2**–**5*) have made WNV infections a public health concern.

The epidemiology of WNV in Europe differs from that in America and has only been associated with nonrecurrent, sporadic outbreaks. The reasons for this difference are controversial; it may be due to environmental factors, reservoirs, or even mosquito vectors. In Spain, neither equine nor human WNV cases have been reported. However, some human serosurveys that used hemagglutination inhibition suggested that WNV or closely related flaviviruses circulated during the 1970s in the Ebro delta and areas in Spain (*6**,**7*). The Ebro delta, a wetland in Catalonia, in the northeast of Spain, is a stopping-off point for birds migrating between regions of Africa and Europe where different WNV vectors and reservoirs have been identified. The delta could be considered a high-risk area for WNV and other arthropodborne virus infections.

To evaluate WNV seroprevalence in the human population of the Ebro delta, a survey was conducted in 2001. After obtaining informed consent, 992 serum samples were obtained from inhabitants of the area. The population studied was representative of the whole area and was stratified by sex and age.

Anti-WNV immunoglobulin G (IgG) antibodies were determined by using an in-house indirect enzyme-linked immunosorbent assay (ELISA), as previously described (*8*). Results were classified as the sample absorbance/positive control absorbance ratio. Samples showing ratio values >0.2 were tested for WNV IgG and IgM by using an indirect and a μ-chain capture ELISA, respectively (Focus Technologies, Cypress, CA, USA), and an in-house microneutralization test.

For the microneutralization test, samples were tested in duplicate and assayed twice. Twofold dilutions (25 μL) of the samples (1:16–1:256 dilutions) were assayed by using 100 TCID_50_ (50% tissue culture infectious dose) of West Nile Eg-101 reference strain in 96-well tissue culture plates with Vero cells and after 7 days of incubation at 37°C and 5% CO_2_.

Thirty-eight samples showed IgG ratios >0.2 by the in-house ELISA. Of these, 12 showed WNV IgG, and 1 was positive for WNV IgM and IgG, according to the Focus assays. Two samples showed positive neutralizing activity, with titers of 32 and 256. The highest titer was shown by the sample that yielded positive levels of both IgM and IgG in the ELISA, which suggests recent WNV infection.

Anti-WNV IgG was more often detected in participants in the 20- to 29-year age group (odds ratio [OR] 4.23, 95% confidence interval [CI] 1.04–16.02, p = 0.03) and in persons who reported frequent mosquito bites (OR 8.62, 95% CI 0.44–169, p = 0.08). IgG-positive persons were equally divided by sex. No significant differences were found between antibody-positive or antibody-negative persons with respect to their profession, place of occupation, current residence, time in current residence, outdoor activities, use of insecticides and repellents, or symptoms related to WNV infection.

No symptoms related to WNV infection were reported by the IgM/IgG-positive participant, who was 31 years of age, was born in the area, worked outdoors, and was frequently bitten by mosquitoes. He also reported travel to Cuba 1 year earlier, but he had not been vaccinated against flavivirus, and serologic test results for dengue were negative.

The other IgG- and neutralizing antibody–positive participant was 45 years of age and was born and works in the area. He had never traveled abroad or been vaccinated against flavivirus. He reported a 4-day fever of unknown origin during the summer 1 or 2 years before the study. He often fishes in the areas and is frequently bitten by mosquitoes.

In conclusion, the study found evidence of recent WNV infections in humans living in the Ebro delta, where previous flavivirus circulation has been suggested by Lozano and Filipe (*6*). IgG-positive results not confirmed by neutralization could be due to cross-reactive antibodies induced by other flavivirus infections or vaccinations (*9**,**10*). The probable WNV infection described was asymptomatic, as occurs in ≈80% of cases. Other WNV infections in the area may have remained undetected, including neuroinvasive cases. Intensified research and surveillance in this area will help determine and refine thresholds for public health interventions.
