# Correction: Vol. 12, No. 7

**DOI:** 10.3201/eid1208.C41208

**Published:** 2006-08

**Keywords:** Correction: Vol. 12, No. 7, erratum, correction, Domingo Bofill

In "Human West Nile Virus Infection, Catalonia, Spain" by Domingo Bofill et al., an error occurred in the final paragraph of the article. The sentence incorrectly states that 20% of cases of West Nile virus infection are asymptomatic. The sentence should read "The probable WNV infection described was asymptomatic, as occurs in ≈80% of cases."

The corrected text appears in the online article at http://wwwnc.cdc.gov/eid/article/12/7/06-0164_article.htm.

We regret any confusion these errors may have caused.

